# Psychometric Properties and Validity of the Screen for Child Anxiety Related Emotional Disorders: Parent Version (SCARED-P) in an Early Childhood Sample

**DOI:** 10.1177/10731911231225203

**Published:** 2024-01-23

**Authors:** Bethan Scoberg, Christopher Hobson, Stephanie van Goozen

**Affiliations:** 1 Cardiff University, UK

**Keywords:** Screen For Child Anxiety Related Emotional Disorders, anxiety disorder, psychometric, child development, early childhood

## Abstract

The Screen for Child Anxiety Related Emotional Disorders: Parent Version (SCARED-P) was originally developed for use in middle childhood and adolescence. The present study examined the psychometric properties and validity of the SCARED-P in an early childhood sample (predominantly aged 4–7 years). The 41-item version of the SCARED-P was administered to the parents of 233 children (mean age = 6.31 years, *SD* = 1.08; females = 34.3%). Confirmatory factor analysis provided mixed support for the original five-factor model of the SCARED-P. The SCARED-P demonstrated good to excellent internal consistency (total α = .94, subscale α = .68–.89), and good construct validity with the Child Behavior Checklist, Strengths and Difficulties Questionnaire, and the Developmental and Well-being Assessment. These findings indicate overall initial support for the SCARED-P’s utility as a measure of anxiety in early childhood, but further psychometric and validation studies are needed in larger community-based samples of young children.

Anxiety can be defined as a feeling of unease, such as worry or fear, that can range from mild to severe. It is a feeling universally experienced and can be adaptive in facilitating danger avoidance (Beesdo et al., 2009). According to the *Diagnostic and Statistical Manual of Mental Disorders* (5th ed.; *DSM-5*; American Psychiatric Association [APA], 2013), anxiety becomes disordered when it has been present for at least 6 months, is associated with a set number of common anxiety symptoms (e.g., edginess or restlessness, irritability, difficulty sleeping), is difficult to control, and causes significant distress or impairment of daily functioning. Anxiety disorders are one of the most common childhood psychological difficulties (Hirshfeld-Becker et al., 2010), with an estimated prevalence of between 9% and 32% across childhood and adolescence (Creswell et al., 2014). It is possible that anxiety rates in younger children are underestimated, due in part to symptoms not being organized into clear and traditionally recognizable patterns that would lead to a diagnosis (Whalen et al., 2017).

Anxiety disorders in childhood are predictors of later adolescent or adult anxiety disorders, major depression, substance abuse, and educational underachievement in adulthood, especially when the childhood anxiety has been poorly managed (Benjamin et al., 2013; Isolan et al., 2011). Hence, the identification and treatment of anxiety during the early stages of childhood are important topics of preventive clinical psychology and psychiatric research.

Although developmental considerations are necessary when considering the clinical significance of anxiety symptoms, the *DSM-5* (APA, 2013) allows for the diagnosis of several anxiety-related disorders in infancy and early childhood. However, most childhood anxiety questionnaire measures have been developed and validated for older children, including the Multidimensional Anxiety Scale for Children (MASC; March et al., 1997), the State-Trait Anxiety Inventory for Children (STAIC; Spielberger et al., 1973), and the Screen for Child Anxiety Related Emotional Disorders (SCARED; Birmaher et al., 1997). Relevant scales that have been developed and validated in both early and later phases of childhood include the Child Behavior Checklist (CBCL; Achenbach, 1991) and the Strengths and Difficulties Questionnaire (SDQ; Goodman, 2001). However, these two widely used questionnaires both only have one anxiety-related subscale which does not assess for a wide range of anxiety disorder symptoms. Langley et al.’s (2002) review summarized measures of anxiety in childhood. Their review did not include any measure for anxiety in children younger than the age of 6, aside from a school refusal measure for children aged older than 5 which was limited in capturing the breadth of anxiety symptoms that a young child might experience.

Measurement of child anxiety is typically through child, parent, or teacher report, or a combination of the three. For younger children, although they are able to report on basic symptomology through developmentally appropriate measures, their reports of abstract or complex symptomology, or levels of impairment, will often present validity concerns (Luby et al., 2007). Therefore, parents and/or teachers play a more central role in the assessment of any symptoms of impairment in younger children than among older children.

The current study is focused on one of the aforementioned anxiety questionnaire measures for children, the SCARED, which was developed by Birmaher et al. (1997). The SCARED was originally designed to screen for anxiety disorders in children aged 8 to 18 years and includes parallel parent and child versions. The parent and child versions of the SCARED show moderate agreement (Runyon et al., 2018), making it a useful tool when child data are difficult to obtain due to such issues as cognitive impairment, oppositionality, lack of child availability, or for children who do not yet have the required cognitive abilities to complete such a measure. The scale consists of 41 items comprising five factors: panic/somatic, generalized anxiety, separation anxiety, social phobia, and school phobia. When the measure was first created, the first four factors corresponded to its *Diagnostic and Statistical Manual of Mental Disorders* (4th ed.; *DSM-IV*; APA, 1994) counterpart diagnoses (Birmaher et al., 1997). This has been complicated somewhat by the publication of *DSM-5*, where changes were made to operational definitions of the disorders. Despite this, Chan and Leung (2015) suggest the original version of the SCARED continues to be appropriate for assessing child and adolescent anxiety symptoms, as the core features related to the classification of anxiety disorders remain unchanged.

The SCARED has been well validated in middle childhood and adolescence (Runyon et al., 2018). Runyon et al.’s (2018) meta-analysis analyzed 65 studies and concluded that the internal consistencies for both the parent (SCARED-P) and child versions were excellent. The tool has also been well validated cross-culturally (the Netherlands: Hale et al., 2005; Brazil: Isolan et al., 2011; China: Su et al., 2008). Of note, Hale et al.’s (2011) meta-analyses of the SCARED found that the original five-factor structure was supported in cross-cultural samples including Europe, the United States, South Africa, and China.

Runyon et al. (2018) identified that of the 65 studies that they analyzed using the SCARED, most used samples of children aged 8 and above. One study included in their review utilized the SCARED with children aged 6 years (Weitkamp et al., 2010) and eight studies included children aged 7 and above. Of these studies, no limitations were raised to highlight difficulties in utilizing the measure with a younger than originally intended sample. Similarly, Langley et al. (2004) concluded that the Child Anxiety Impact Scale–Parent version (a narrower measure of anxiety than the SCARED, covering school, social, and home/family anxiety symptoms) functioned similarly well in the 4- to 8-year-old subsection of their sample as it did with their older child sample (up to 17 years old).

The robust validation of the SCARED in middle childhood, paired with its well-established factor structure, makes the measure a strong contender for its use in younger children. In a younger age group, though, it is likely that only the parent version will be valid given the range of symptoms covered in the SCARED. Thus, there is justification to formally investigate the psychometric properties and validity of the SCARED: parent version (SCARED-P) in an early childhood sample. If the SCARED-P were found to be valid in early childhood, this could have helpful clinical implications (e.g., to aid screening for anxiety disorders in early years clinical or education settings) and research implications (e.g., tracking anxiety over time in longitudinal studies using a consistent measure from early childhood onward). It would also negate the need to develop entirely new broad questionnaire measures of anxiety in early childhood.

In summary, although the SCARED-P was originally validated in children 8 years and older, a few studies have shown that it is also a valid measure in children as young as 6. To the authors’ knowledge, no previous study has explicitly validated the SCARED-P in a predominantly early childhood sample. Therefore, the aim of the current study was to consider the psychometric properties and validity of the SCARED-P: in an early childhood sample (predominantly children aged 4–7 years).

The specific hypotheses of the study were, first, that the original theoretical five-factor model (Birmaher et al., 1997) of the SCARED-P would be supported in this sample. Second, we hypothesized that the SCARED-P subscales would have good internal consistency. Third, we expected significant positive correlations with similar constructs in validated questionnaire measures (specifically, the SCARED-P Total with the CBCL Anxious/Depressed and SDQ Emotional subscales; the SCARED-P Somatic/Panic subscale with the CBCL Somatic Complaints subscale, the SCARED-P Generalized Anxiety subscale with the CBCL Anxious/Depressed and SDQ Emotional subscales). Fourth, we expected positive correlations with similar constructs in the Development and Well-Being Assessment (DAWBA; specifically, the SCARED-P Total with the DAWBA Generalized Anxiety Disorder score, the SCARED-P Generalized Anxiety subscale with the DAWBA Generalized Anxiety Disorder score; the SCARED-P Separation Anxiety subscale with the DAWBA Separation Anxiety score; and the SCARED-P Social Phobia subscale with the DAWBA Social Anxiety score).

Further exploratory analyses were also planned to consider convergent validity, to assess whether there was a notable difference in the strength of correlations between the SCARED-P Total and internalizing and externalizing behavior on the CBCL (as has been noted in older samples; e.g., Su et al., 2008). Further analyses were also planned to assess whether certain SCARED-P items were redundant in younger children based on comparing responses of parents of 4- to 5-year-old children with responses of parents of children aged 6 years and older.

## Method

### Participants

Two hundred and thirty-three parent–child dyads participated in this study, having been referred by teachers to the Neurodevelopment Assessment Unit (NDAU) at Cardiff University. Participating children were aged between 4 and 9 years (*M* = 6.31 *SD* = 1.08; females = 34.3%). NDAU recruit children experiencing emotional and/or behavioral difficulties at school, and all participants included in this sample were seen at NDAU between October 2017 and March 2020. The majority of the sample (*N* = 222) were in the 4- to 7-year age bracket as per the NDAU referral criteria. However, as the NDAU also provides a feedback report containing educational psychology advice based on some of its normed measures, due to waiting list delays, a small number of children above 7 years old were also included in the sample on the basis of need (age 8, *N* = 10; age 9; *N* = 1). The sample was largely of White British ethnicity (81.5%). Ethical approval was granted for the project (EC.16.10.11.4592GRA5).

### Measures

The *SCARED: Parent version* (SCARED-P; Birmaher et al., 1997) is a 41-item measure of child anxiety. The SCARED-P includes five factors: panic/somatic (13 items; e.g., “When my child feels frightened, it is hard for him or her to breathe”), generalized anxiety (nine items; e.g., “My child worries about things working out for him or her”), separation anxiety (eight items; e.g., “My child gets scared if he or she sleeps away from home”), social phobia (seven items; e.g., “My child feels nervous with people he or she doesn’t know well”), and school phobia (four items; e.g., “My child gets stomach aches at school”). Severity of symptoms is rated for the past 3 months using a 3-point scale (0 = *not true or hardly ever true*; 1 = *sometimes true*; 2 = *true or often true*). Scores range from 0 to 82, and higher scores reflect higher levels of anxiety.

The SDQ (Goodman, 2001) is a parental and teacher-based assessment tool which enables assessment of internalizing and externalizing difficulties within children. Parental assessments only were included in this study. The questionnaire is divided into five subscales: emotional problems, hyperactivity, conduct problems, peer problems, and prosocial scales. Due to the specific hypotheses and focus of this study, the total score, emotional problems subscale, and peer problems subscale (both five items) were analyzed.

The *CBCL* (Achenbach, 1991) is a 118-item parent measure for assessing child emotional and behavioral problems in children aged 4 to 18 years. The CBCL produces a total score that ranges between 0 and 240; lower scores indicate poorer functioning. The total score and four subscales were utilized in this study, specifically, the CBCL anxious/depressed raw score (13 items), the CBCL somatic complaints raw score (11 items), and the Internalizing and Externalizing subscales (these are larger subscales that incorporate several subscales to generate a score for Internalizing and Externalizing problems, and were used to assess convergent validity).

The DAWBA (Goodman et al., 2000) interview was designed to generate International Classification of Diseases, 10th Revision (ICD-10; World Health Organization, 2016) and *DSM-IV* diagnoses. Due to the specific hypotheses and focus of this study, the separation anxiety, social anxiety, and generalized anxiety (GAD) symptom counts were included in the analyses.

### Procedure

Parents provided written informed consent for data to be used for research purposes. The measures included in the current study are a small subset of a larger battery of child and parent measures. Parent–child dyads visited the NDAU to complete an assessment by trained postdoctoral researchers and postgraduate students usually over two testing sessions. Children completed their measures while parents were interviewed (including the DAWBA) and completed a battery of questionnaire measures (including the CBCL, SDQ, and SCARED-P).

### Data Analysis

To test the factor structure of the SCARED-P, both a one-factor model and a five-factor model were tested using confirmatory factor analysis (CFA). For the CFA, the structural equation modeling program Mplus Version 8.6 was used (Muthén & Muthén, 2007). As the data did not justify the assumption of multivariate normality, the weighted least square mean and variance (WLSMV) adjusted estimator was employed, as this is an appropriate estimator for ordinal data (T. Brown, 2006). To evaluate the fit of the model, we relied on the following indices: χ^2^, with χ^2^ evaluated relative to degrees of freedom (χ^2^/*df*), with <2 indicating good model fit (Mueller, 1999); comparative fit index (CFI), with ρ > .90 indicating an acceptable model fit (Bentler, 1990); root mean square error of approximation (RMSEA), with <.08 indicating an acceptable fit and <.06 indicating a good fit (M. W. Brown & Cudeck, 1993); and standardized root mean square residual (SRMR), with <.08 representing an acceptable fit (Hu & Bentler, 1998).

IBM SPSS Statistics Version 25 was used for the remaining analyses. Cronbach’s α coefficients were calculated to evaluate the internal consistency of the SCARED-P total and subscales. Bivariate Pearson correlations were utilized to assess the relationships between the SCARED-P and the demographic variables (age, gender) and related measures (questionnaires and diagnostic interview). Chi-square test of independence was utilized to understand differences between responses of parents with younger (4- to 5-year-olds) and older (6 years and older) children.

Where the data utilized did not meet the assumptions of normal distribution for the usage of parametric statistics, transformations were applied. Data were defined as non-normal when the skewness statistic was two times greater than the standard error of skew. Three transformations were utilized where appropriate: logarithmic, square root, and reciprocal. The transformation which reduced the skewness statistic most significantly was retained for later analysis. Following the data transformation, no outliers were identified within the data.

Of a potential 255 subjects, 22 were excluded as they had >10% incomplete questionnaire data on the SCARED-P measure. For subjects who had <10% missing questionnaire data on the SCARED-P measure, the missing data were handled using case mean imputation.

## Results

Descriptive statistics for the sample are reported in Table 1 for questionnaire and diagnostic interview data. Bivariate correlations between variables and age are reported in Table 2.

**Table 1.** Descriptive Statistics of Measures.

Measures	*M* (*SD*)	Range
SCARED-P total	20.03 (14.4)	0–70
SCARED-P somatic/panic	3.18 (4.18)	0–24
SCARED-P generalized anxiety	5.13 (4.21)	0–17
SCARED-P separation anxiety	5.25 (4.03)	0–16
SCARED-P social phobia	5.03 (3.93)	0–14
SCARED-P school phobia	1.45 (1.72)	0–7
SDQ total	18.4 (6.9)	2–34
SDQ emotional (parent)	3.63 (2.7)	0–10
SDQ peer (parent)	3.11 (2.3)	0–9
CBCL total	58.7 (32.2)	0–147
CBCL anxious/depressive raw	5.72 (4.5)	0–22
CBCL internalizing	14.68 (11.0)	0–51
CBCL externalizing	19.61 (12.2)	0–50
CBCL anxious raw	6.33 (5.6)	0–42
CBCL somatic complaints	2.92 (3.2)	0–14
DAWBA separation anxiety	1.29 (1.9)	0–8
DAWBA social anxiety	1.03 (1.8)	0–6
DAWBA GAD	0.69 (1.7)	0–6

*Note.* SCARED-P = Screen for Child Anxiety Related Emotional Disorders: Parent Version; SDQ = Strengths and Difficulties Questionnaire; CBCL = Child Behavior Checklist; DAWBA = Development and Well-Being Assessment; GAD = Generalized Anxiety.

**Table 2.** Bivariate Pearson Correlations of SCARED-P Subscales, and Age.

Measure	1	2	3	4	5	6	7
1. Age	1	.13	.12	.20**	.05	.02	.10
2. SCARED-P total		1	.82**	.87**	.82**	.71**	.68**
3. SCARED-P somatic/panic			1	.65**	.60**	.40**	.53**
4. SCARED-P generalized anxiety				1	.61**	.54**	.50**
5. SCARED-P separation anxiety					1	.45**	.55**
6. SCARED-P social phobia						1	.37**
7. SCARED-P school							1

*Note.* Age recorded in months. SCARED-P = Screen for Child Anxiety Related Emotional Disorders–Parent version.

** *p* < .001.

### Relationship Between SCARED-P, Age, and Gender

Correlations indicated that older age was related to higher scores on the SCARED-P Generalized Anxiety subscale, but not the SCARED-P total score or any other subscale scores. Independent *t* tests indicated that there were no significant gender differences in the SCARED-P total or subscale scores: Total, *t*(231) = 1.48, *p* = .14; Panic/Somatic, *t*(119.47) = 1.45, *p* = .15; Generalized Anxiety, *t*(139.90) = 1.65, *p* = .10; Separation Anxiety, *t*(231) = 0.48, *p* = .63; Social Phobia, *t*(231) = 1.33, *p* = .18; and School Phobia, *t*(231) = 0.58, *p* = .56.

### Confirmatory Factor Analysis

**Figure 1:**
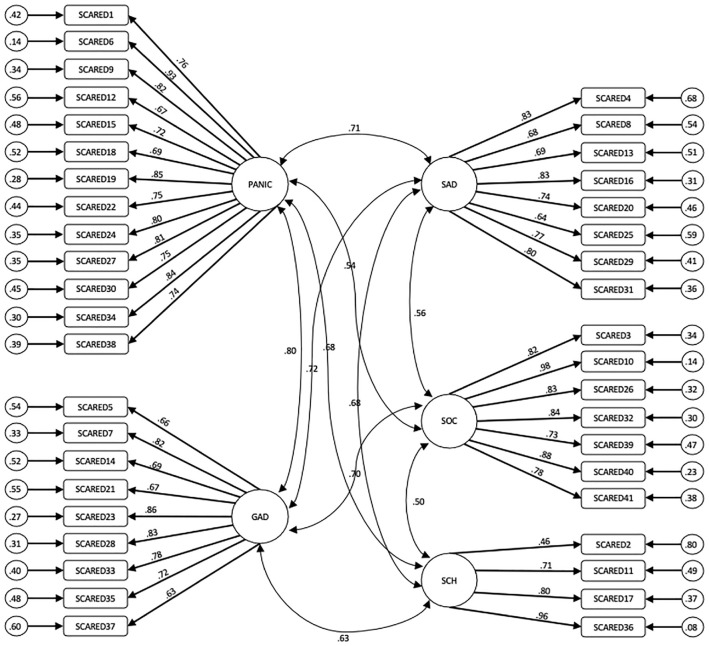
The Five-Factor Model of the SCARED-P. *Note.* Panic = SCARED-P Somatic/panic subscale; SAD = SCARED-P Separation anxiety subscale, SOC = SCARED-P Social Phobia subscale; GAD = SCARED-P Generalized Anxiety subscale; SCH = SCARED-P School Phobia subscale. Smaller circles denote residual variance.

Our first hypothesis was that the original theoretical five-factor model (Birmaher et al., 1997) of the SCARED-P would be supported by the data. The five-factor model reported by Birmaher et al. (1997) had a better fit than the one-factor model. The one-factor model fit indices were: χ^2^(1,882.67)*/df* (779)= 2.4, *p <* .0001, CFI = .83, RMSEA = .078. For the five-factor model, the three most commonly reported model fit indices in the literature (χ^2^/*df*, CFI, RMSEA) indicated an adequate fit: χ^2^(1,259.30)*/df* (769) = 1.63, *p <* .0001, CFI = .92, RMSEA = .052. However, the SRMR model fit indices did not indicate a good fit (SRMR = .097). The parameter estimates and standard errors are shown in Figure 1. As shown by the parameter estimates, all but one reached >.60, indicating that the items loaded adequately on each factor.

### Internal Consistency

Our second hypothesis was that the SCARED-P subscales would have satisfactory internal consistency. The internal consistency of the SCARED-P total score and subscales was measured by calculating Cronbach α coefficients. The coefficient α values were .94 for the total score, .87 for somatic/panic, .86 for generalized anxiety, .83 for separation anxiety, .89 for social phobia, and .68 for school phobia.

### Construct Validity

Our third hypothesis was that the data would support the SCARED-P’s construct validity in terms of expected correlations with similar constructs in questionnaire measures (SDQ and CBCL) and in a diagnostic interview measure (DAWBA). All 233 subjects were also evaluated using the CBCL and SDQ (parent version). Total scores and relevant subscales were used to establish construct validity both of the SCARED-P total, and the SCARED-P subscales with corresponding subscales from the CBCL and SDQ questionnaires. Construct validity of the SCARED-P was also assessed by Pearson correlations with the DAWBA interview symptom counts corresponding to relevant *DSM* diagnoses. All predicted relationships (in bold in Table 3) were found to be positively and significantly correlated (*p <* .001).

**Table 3.** Correlations Between SCARED-P Total and Subscales With Theoretically Relevant Measures of Anxiety in This Child Sample.

Measure	SCARED-P total and subscales					
	Total	Somatic/panic	Generalized anxiety	Separation anxiety	Social phobia	School phobia
CBCL total	.42 **	.43**	.28**	.38**	.23**	.34**
CBCL anxious/depressed	.67**	.54**	.61**	.54**	.47**	.40**
CBCL somatic complaints	.36**	.37**	.24**	.31**	.17	.43**
SDQ total	.41 **	.39**	.30**	.37**	.19**	.39**
SDQ emotional	**.72** **	.56**	.61**	.56**	.55**	.47**
SDQ peer	.15	.22**	.10	.15	.02	.26 **
DAWBA separation anxiety	.61**	.49**	.42**	.67**	.32**	.42**
DAWBA social anxiety	.40**	.27**	.34**	.24**	.43**	.22**
DAWBA GAD	**.46** **	.35**	.42**	.34**	.29**	.31**

*Note.* Hypothesized correlations are in bold. SCARED = Screen for Child Anxiety Related Emotional Disorders; CBCL = Child Behavior Checklist; SDQ = Strengths and Difficulties Questionnaire; DAWBA = Development and Well-Being Assessment; GAD = Generalized Anxiety.

** *p* < .001.

### Further Exploratory Analyses

#### Convergent and Divergent Validity

The SCARED-P total correlated significantly and positively with the parents’ CBCL internalizing score (*r* = .54, *p <* .001), where correlations above *r* = .50 are suggested as an acceptable level of convergent validity (Carlson & Herdman, 2012). With regard to divergent validity, the SCARED-P total showed a significant, but lower strength correlation with the externalizing score (*r* = .20, *p <* .01). These findings were in line with other convergent validity analyses of the SCARED (e.g., Monga et al., 2000; Su et al., 2008).

#### Age Related Item Redundancy

We undertook further exploratory analyses to consider whether certain SCARED-P items were redundant based on comparing responses of parents of 4- to 5-year-old children to parents of children aged 6 years and older. First, inter-item correlations were calculated to assess item redundancy across the sample, by examining the extent to which scores on one item of the SCARED-P are related to scores on all other items of the SCARED-P. Items had a mean inter-item correlation of 0.28, within the ideal 0.2 to 0.4 range (Piedmont, 2014). Next, to assess for redundant items by age, the sample was split into 4- to 5-year-olds (*N* = 93) and above 6-year-olds (*N* = 140). Responses to the 41 items of the SCARED-P were categorized as 0 (indicating the item was not endorsed by parents; i.e., scored as “not true”) or 1 (indicating a parent scored the item as either “somewhat true” or “very true”). A chi-square test of independence showed no significant difference between the responses of parents of 4- to 5-year-olds and parents of above 6-year-olds on 40 of the 41 SCARED-P items. Item 33 (“My child worries about what’s going to happen in the future”) was significantly different (*p* = .008) between the two age groups in that parents of older children tended to be more likely to endorse this item.

## Discussion

The field of childhood anxiety is limited by the lack of specific questionnaire measures of anxiety suitable for early childhood. Therefore, this study sought to add to the research base by examining the psychometric properties of the SCARED-P in an early childhood sample. The results will now be discussed as they relate to each of the hypotheses.

The first hypothesis was that the original theoretical five-factor model (Birmaher et al., 1997) of the SCARED, as found in older children, would be supported in this early childhood sample. Our analysis indicated mixed support for the five-factor model. Three model fit indices (χ^2^*/df*, CFI, and RMSEA) demonstrated acceptable model fit, and one model fit index (SRMR) demonstrated inadequate model fit. It is important to note that other psychometric studies concerning the SCARED have found mixed model fits for the original five-factor structure in older community child samples (e.g., Boyd et al., 2003; Essau et al., 2013). The model fit index that suggested an inadequate model fit, SRMR, has not been consistently reported in other studies conducting CFA on the SCARED (Essau et al., 2013; Su et al., 2008). Unlike the other model fit indices, the SRMR is calculated based on residual (unexplained) variance. Indeed, there was a relatively high level of residual variance in some of the variables in the model, meaning that there may be further relationships existing within the data that are not explained by the original five-factor model.

It is noted that the sample was made up of children referred by schools to a research study due to emotional and behavioral difficulties (more akin to a “clinical sample” rather than a “community” sample) which might explain larger than expected cross-loading of items due to increased comorbidity. Several other SCARED-P validation samples have utilized community samples, which may explain the differing results of this study (Hale et al., 2005; Isolan et al., 2011; Su et al., 2008). Despite the overall model fit findings, the CFA did demonstrate that items loaded adequately onto each factor. Furthermore, consistent with previous research, there were correlations both among factors, and between the SCARED-P total and all factors. Taken together, the findings not only indicate mixed support for the five-factor structure of the SCARED-P in an early childhood sample, but also suggest more research is required in early childhood community samples.

In line with the second hypothesis, the internal consistency of the SCARED-P total score was found to be excellent (α = .94), with subscales indicating good to excellent levels. A high coefficient α can be indicative of high item redundancy; however, the average inter-item correlation fell within the advised inter-item correlation range of .2 to .4 (Piedmont, 2014), suggesting item redundancy was not an issue. Within the SCARED-P subscales, the lowest internal consistency was for the school subscale (α = .68). This is consistent with other studies in older children (Essau et al., 2013; Isolan et al., 2011; Su et al., 2008) and may be due in part to the small number of items in this subscale (*N* = 4). The overall picture is that the SCARED-P and its subscales demonstrated sufficient reliability in this predominantly early childhood sample.

The third and fourth hypotheses were that the data would support the SCARED-P’s construct validity across related questionnaire measures and a diagnostic interview. The hypothesized relationships between the CBCL, SDQ, and SCARED-P were found to be significant and in the expected directions. The related subscales from the DAWBA diagnostic interview correlated with the corresponding subscales of the SCARED-P. Taken together, these results demonstrate SCARED-P’s satisfactory construct validity with related questionnaire and interview measures. Furthermore, in line with Monga et al. (2000), which utilized the CBCL as a comparison measure, the SCARED-P also showed good convergent and divergent validity in terms of its relationships with the CBCL internalizing and externalizing scores.

In the current sample, there was no relationship between gender and the SCARED-P total or subscales, a finding that is not in line with other studies, which have typically found females to exhibit higher total anxiety and/or on higher scores on particular subscales (Birmaher et al., 1997; Hale et al., 2005, 2011). Reasons for this disparity could be the nature of our sample being more akin to a clinical sample, and that gender differences in anxiety have typically been reported in community samples.

In our exploratory analyses, there was little evidence to suggest that a significant number of items were not relevant for the younger age group of 4- to 5-year-olds. A chi-square test of independence showed that there were no significant differences in item endorsement between younger and older age groups for 40 of the 41 items, suggesting that the vast majority of the SCARED-P items were interpreted and answered in a similar way irrespective of whether the child was toward the younger (4- to 5-year-olds) or older (6+ years) end of the sample, adding weight to the SCARED-P’s utility as a measure for anxiety in early childhood. These findings should be interpreted with caution due to the relatively modest sample size.

As outlined, research in the field of early childhood anxiety is important due to its clinical and research implications. A strength of this study is that it contributes to the early childhood anxiety research base, which is important given the evidence suggesting anxiety disorders in childhood are strong predictors of later adolescent or adult anxiety disorders (Benjamin et al., 2013; Isolan et al., 2011). Validation of a psychometric measure can aid in the identification of children in need of early intervention work. The sample utilized in this study is particularly relevant when considering children who might be most in need of such a measure, as all children were referred by their school for identified emotional or behavioral difficulties, therefore making this a clinically relevant sample. Finally, we used a variety of methods to validate the SCARED-P, including related questionnaire measures and a diagnostic interview which increases the robustness of the validation.

There are a number of limitations in relation to the present study which must be taken into consideration when interpreting these results. One limitations relate to the sample utilized in this study which might not represent a typical anxious sample, given that many of the children were likely referred into the study due to concerns about both internalizing and externalizing behavioral problems (and hence the existence of comorbidity). It is unclear whether the reported findings would be generalizable to either “purer” anxiety-specific clinical samples or indeed community samples.

It could be argued that as this sample uses a younger age group than the original paper (Birmaher et al., 1997), an exploratory factor analysis could have been conducted instead of a CFA as an alternative factor structure may have been more appropriate for the symptomology of a younger age group. However, a CFA was considered appropriate as no clear theoretical reason was identified to suggest there would be a different factor structure in younger children. For example, the SCARED-P has been utilized for 6-year-olds with no issues reported (Weitkamp et al., 2010).

We utilized the SCARED-P in its original format and acknowledge that the wording of the questions may not be applicable to all younger children. For example, questions relating to school may not be relevant for all 4-year-olds. We recommend that when administering to younger children a “not applicable” option is added, and scores pro-rated accordingly.

Finally, it was not possible to conduct test–retest reliability statistics, as this sample were seen at just one time point prior to the Covid-19 pandemic (and hence post-pandemic assessments would have been unsuitable for test–retest reliability due to the occurrence of a significant societal event having a likely impact upon children’s anxiety levels). Future studies would benefit from assessing the test–retest reliability to further validate the measure in early childhood samples.

Having taken into account the strengths and limitations of this research study, given the paucity of anxiety measures for younger children, our findings indicate that the SCARED-P may be valuable when considering early identification for childhood anxiety. It is noted that the evaluation of interventions for early childhood anxiety is a relatively recent area of investigation, and research indicates that interventions for younger children may look different to the recommended course of treatment for older children (Comer et al., 2019). Parents tend to play a more integral role in interventions for early childhood anxiety (Anticich et al., 2012). Therefore, a valid measure for early childhood anxiety that utilizes parent report fits with the overall approach toward early childhood anxiety, with parents seen as both key informants and key agents of change in their young child’s anxiety.

## Conclusion

The overall findings in the present study demonstrate that the SCARED-P appears to be a reliable and valid measure for early childhood anxiety. The factor structure would benefit from further validation in a wider early childhood community sample, to increase the sample size and diversity. As the SCARED-P is easy to administer, cost-effective, and, as it is well validated in older childhood, it can serve as a consistent measure for tracking anxiety over time. Given the impact that early childhood anxiety can have both on day-to-day functioning and longer-term outcomes, the SCARED-P may be a valuable tool for screening younger children for further assessment and intervention.
